# Measurement Opportunities and Challenges With Long-Term Care Residents With Dementia

**DOI:** 10.1093/geroni/igaa057.2807

**Published:** 2020-12-16

**Authors:** Sarah Holmes, Elizabeth Galik, Barbara Resnick

**Affiliations:** 1 University of Maryland School of Pharmacy, Baltimore, Maryland, United States; 2 University of Maryland School of Nursing, Ellicott City, Maryland, United States; 3 University of Maryland School of Nursing, Baltimore, Maryland, United States

## Abstract

Measuring clinical outcomes among older adults with moderate to severe dementia present practical and psychometric challenges. Proxy report surveys, direct observations, chart abstraction, and objective measures, such as actigraphy are frequently used with this population. The purpose of this paper is to describe issues related to measurement in the FBFC trial. Challenges to actigraphy included the resistance of residents and removal of devices, inability to detect limited movement and/or the type of activity performed. The combined use of the three measures to optimize understanding of physical activity will be addressed. Behavioral outcome measures included the Cornell Scale for Depression in Dementia, the Cohen Mansfield Agitation Inventory, and the Resistiveness to Care Scale. Despite the use of these multiple measures, limited behaviors were identified due to timing of assessments and items included within the measures. Optimal ways in which to capture behavioral symptoms will be reviewed.

